# Supplementary material for: Computationally Selected Multivalent HIV-1 Subtype C Vaccine Protects Against Heterologous SHIV Challenge
Source: Vaccines (Basel). 2025 Feb 24;13(3):231. doi: 10.3390/vaccines13030231 (PMC11945704; doi:10.3390/vaccines13030231)
Supplement: Supplementary file 1 [file vaccines-13-00231-s001.zip › vaccines-3398908-supplementary.pdf]

Supplementary Information. Computationally selected multivalent HIV-1 subtype C vaccine protects against heterologous SHIV challenge.

Computationally Selected Multivalent HIV-1 Subtype C Vaccine Protects Against Heterologous SHIV Challenge

Dieter Mielke [1,2,3,†], Marina Tuyishime [1,2,3,†], Natasha S. Kelkar [4,†], Yunfei Wang [1,†], Robert Parks [1], Sampa Santra [5], Wes Rountree [1], LaTonya D. Williams [1,2,3], Tiffany Peters [1,2,3], Nathan Eisel [1,2,3], Sheetal Sawant [1,2,3], Lu Zhang [1,2,3], Derrick Goodman [1,2,3], Shalini Jha [3], Adam Zalaquett [3], Pratamesh Ramasubramanian [3], Sherry Stanfield-Oakley [3], Gary Matyas [6], Zoltan Beck [6], Mangala Rao [6], Julie Ake [6], Thomas N. Denny [1], David C. Montefiori [1,3], Margaret E. Ackerman [4,7], Lawrence Corey [8], Georgia D. Tomaras [1,2,3,9,10], Bette T. Korber [11,12], Barton F. Haynes [1], Xiaoying Shen [1,2,3,*] and Guido Ferrari [1,2,3,10,*]

[1] Duke Human Vaccine Institute, Duke University, Durham, NC 27710, USA; dieter.mielke@duke.edu (D.M.); marina.tuyishime@duke.edu (M.T.); yunfei.wang@duke.edu (Y.W.); rob.parks@duke.edu (R.P.); wes.rountree@duke.edu (W.R.); latonya.williams@duke.edu (L.D.W.); tdpeters@email.unc.edu (T.P.); neisel@carolinas.vcom.edu (N.E.); sheetal.sawant@duke.edu (S.S.); lu.zhang809@duke.edu (L.Z.); derrick.goodman@duke.edu (D.G.); thomas.denny@duke.edu (T.N.D.); david.montefiori@duke.edu (D.C.M.); gdt@duke.edu (G.D.T.); barton.haynes@duke.edu (B.F.H.)

[2] Duke Center for Human Systems Immunology, Duke University, Durham, NC 27701, USA

[3] Department of Surgery, Duke University, Durham, NC 27710, USA; shalini.jha@duke.edu (S.J.); adam.zalaquett@duke.edu (A.Z.); pratamesh.ramasubramanian@gmail.com (P.R.); sherry.oakley@duke.edu (S.S.-O.)

[4] Department of Microbiology and Immunology, Geisel School of Medicine at Dartmouth, Dartmouth College, Hanover, NH 03756, USA; natasha.s.kelkar.gr@dartmouth.edu (N.S.K.); margaret.e.ackerman@dartmouth.edu (M.E.A.)

[5] Beth Israel Deaconess Medical Center, Harvard Medical School, Boston, MA 02215, USA; ssantra@bidmc.harvard.edu

[6] U.S. Military HIV Research Program, Walter Reed Army Institute of Research, Silver Spring, MD 20910, USA; gmatyas@hivresearch.org (G.M.); zoltan.beck@gmail.com (Z.B.); mrao@hivresearch.org (M.R.); jake@hivresearch.org (J.A.)

[7] Thayer School of Engineering, Dartmouth College, Hanover, NH 03755, USA

[8] Vaccine and Infectious Disease Division, Fred Hutchinson Cancer Center, Seattle, WA 98104, USA; lcorey@fredhutch.org

[9] Department of Integrative Immunobiology, Duke University School of Medicine, Durham, NC 27710, USA

[10] Department of Molecular Genetics and Microbiology, Duke University School of Medicine, Durham, NC 27710, USA

[11] T-6: Theoretical Biology and Biophysics, Los Alamos National Laboratory, Los Alamos, NM 87545, USA; btk@lanl.gov

[12] New Mexico Consortium, Los Alamos, NM 87545, USA

[*] Correspondence: sxshen@duke.edu (X.S.); gflmp@duke.edu (G.F.)

[†] These authors contributed equally to the study.

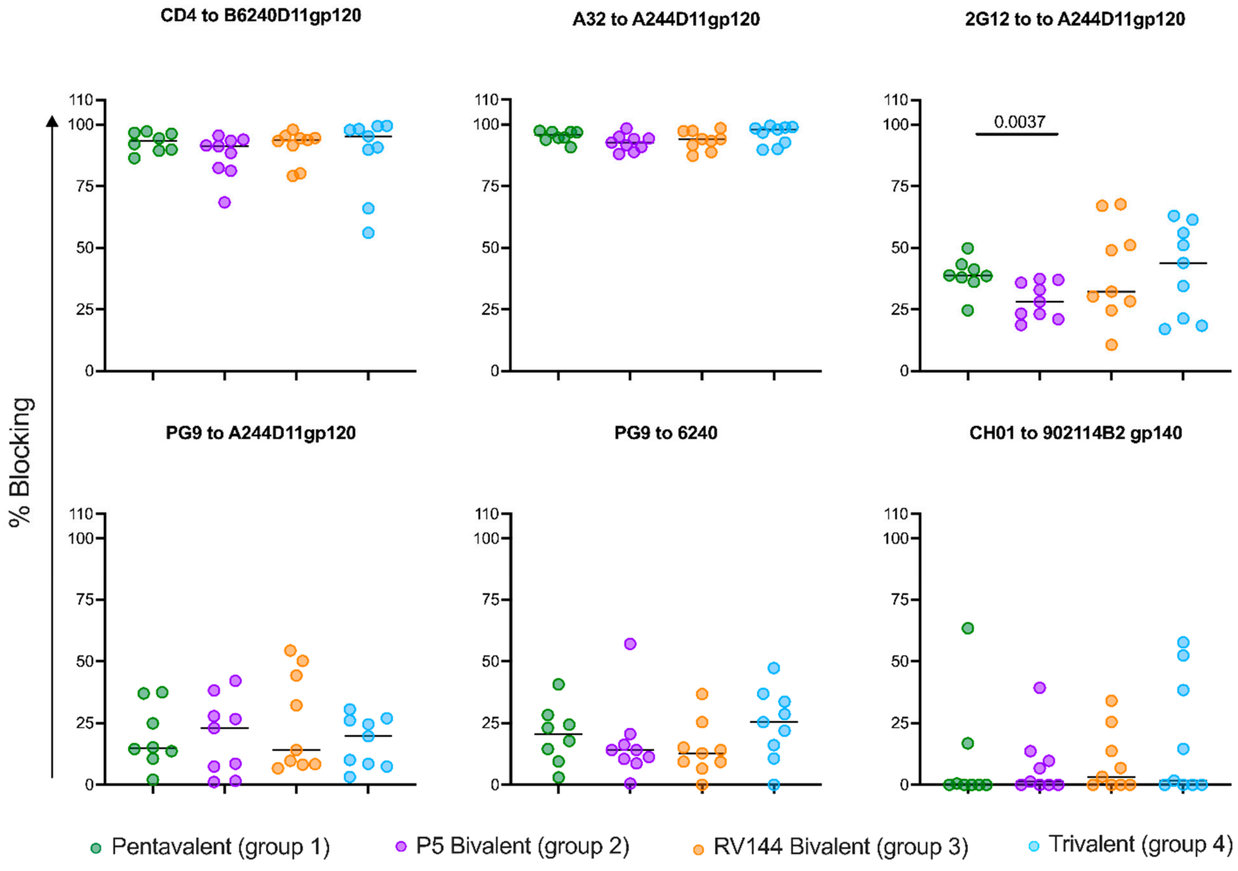

Figure S1. Sera blocking of protein binding to different Envelope gp120 proteins.

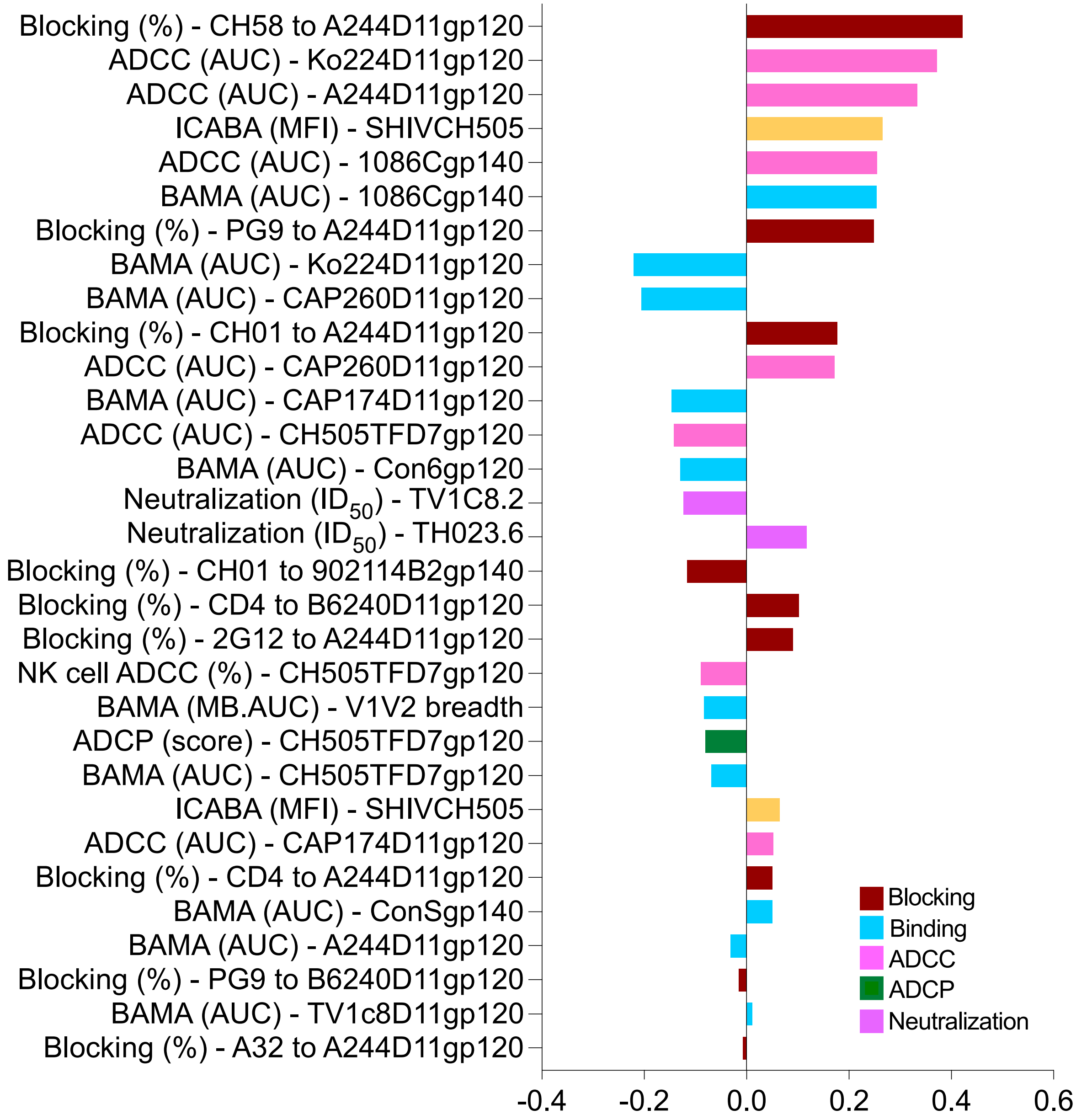

Figure S2. Immune features contributing to PC2. Features are ranked by their relative importance to defining PC2.

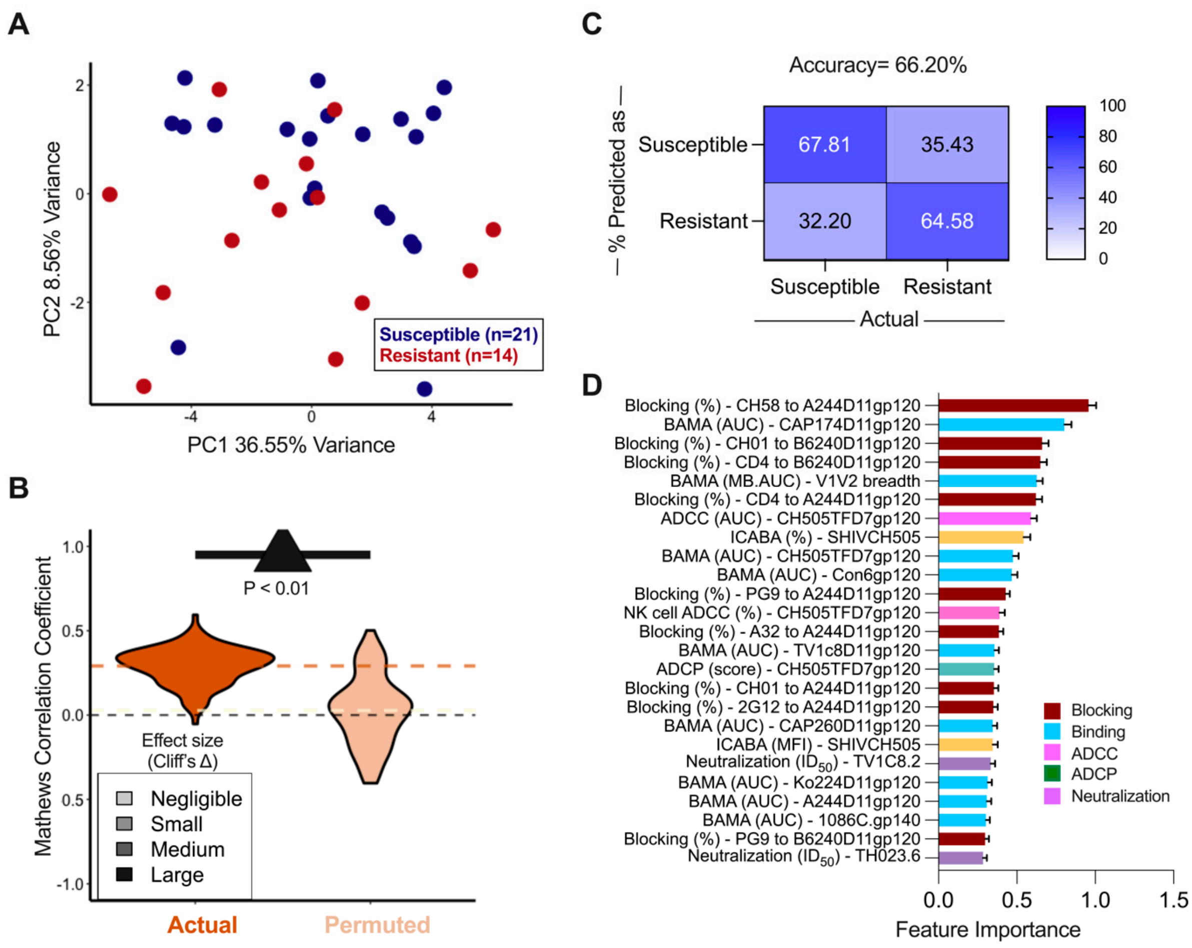

Figure S3. Immune feature profiles can classify animals based on their relative susceptibility to infection. (**A**) Principal component biplot. Animals are represented as dots, with color indicating the susceptibility to infection. Animals that were infected at ≤4 challenges were defined as susceptible (blue, n=21), while animals infected at challenge 5 or that remained uninfected were defined as resistant (red, n=14). (**B**) Accuracy (Matthew's Correlation Coefficient) of relative risk group classification predictions with actual and permuted group labels across modeling replicates. Dotted orange and yellow lines indicate distribution means for actual and permuted datasets and dotted black line indicates performance expected at random (0.0). Statistical significance was defined by Kolmogorov Smirnov Test (inset) and effect size by Cliff's Delta (color). (**C**) Confusion matrix depicting the percentage of animals classified to each relative risk group, and accuracy of classification as determined by the F1 score. (**D**) Average feature importance of the top 25 features that contributed the most in building the classifier across 100 repeats. Error bars represent standard deviation across repeats.

Supplementary tables

Table S1. Raw and FDR-corrected p-values for all pairwise immunological assay assessments.

Assay	Antigen	comparison	raw p-value	False Discovery Rate
ADCP	CH505TFD7gp120	Group 1 vs Group 2	0.1905	0.6387
		Group 1 vs Group 3	0.8148	0.9539
		Group 1 vs Group 4	0.4660	0.8805
		Group 2 vs Group 3	0.0727	0.3864
		Group 2 vs Group 4	**0.0379**	0.2694
		Group 3 vs Group 4	0.7478	0.9263
BAMA	A244D11gp120	Group 1 vs Group 2	**0.0055**	0.0832
		Group 1 vs Group 3	0.5414	0.8805
		Group 1 vs Group 4	0.8884	0.9859
		Group 2 vs Group 3	0.4894	0.8805
		Group 2 vs Group 4	**0.0315**	0.2324
		Group 3 vs Group 4	0.3865	0.7980
	CAP174D11gp120	Group 1 vs Group 2	0.0745	0.3864
		Group 1 vs Group 3	0.0745	0.3864
		Group 1 vs Group 4	0.5414	0.8805
		Group 2 vs Group 3	0.6048	0.9017
		Group 2 vs Group 4	0.1135	0.4842
		Group 3 vs Group 4	**0.0315**	0.2324
	CAP260D11gp120	Group 1 vs Group 2	0.1388	0.5330
		Group 1 vs Group 3	0.0592	0.3669
		Group 1 vs Group 4	0.1672	0.5944
		Group 2 vs Group 3	0.6665	0.9206
		Group 2 vs Group 4	**0.0106**	0.1274
		Group 3 vs Group 4	**0.0056**	0.0832
	CH505TFD7gp120	Group 1 vs Group 2	0.4807	0.8805
		Group 1 vs Group 3	0.0745	0.3864
		Group 1 vs Group 4	0.9626	0.9936
		Group 2 vs Group 3	0.4363	0.8726
		Group 2 vs Group 4	0.3865	0.7980
		Group 3 vs Group 4	0.1359	0.5330
	Ko224D11gp120	Group 1 vs Group 2	0.1996	0.6387
		Group 1 vs Group 3	0.0927	0.4239
		Group 1 vs Group 4	0.2359	0.6563
		Group 2 vs Group 3	0.5457	0.8805
		Group 2 vs Group 4	0.0503	0.3220
		Group 3 vs Group 4	**0.0188**	0.1712
	TV1c8D11gp120	Group 1 vs Group 2	0.6058	0.9017
		Group 1 vs Group 3	0.4807	0.8805
		Group 1 vs Group 4	1.0000	1.0000
		Group 2 vs Group 3	0.2581	0.6859
		Group 2 vs Group 4	0.9314	0.9936
		Group 3 vs Group 4	0.5457	0.8805
	1086Cgp140	Group 1 vs Group 2	0.5414	0.8805
		Group 1 vs Group 3	0.5414	0.8805

		Group 1 vs Group 4	0.0927	0.4239
		Group 2 vs Group 3	0.3865	0.7980
		Group 2 vs Group 4	0.0503	0.3220
		Group 3 vs Group 4	0.2973	0.7048
V1V2 MB-AUC		Group 1 vs Group 2	0.0712	0.3864
		Group 1 vs Group 3	0.2319	0.6563
		Group 1 vs Group 4	0.5350	0.8805
		Group 2 vs Group 3	**0.0055**	0.0832
		Group 2 vs Group 4	1.0000	1.0000
		Group 3 vs Group 4	0.0721	0.3864
Neutralization	TH023.6	Group 1 vs Group 2	**0.0010**	**0.0316**
		Group 1 vs Group 3	0.7430	0.9263
		Group 1 vs Group 4	0.9626	0.9936
		Group 2 vs Group 3	**0.0019**	0.0508
		Group 2 vs Group 4	**0.0315**	0.2324
		Group 3 vs Group 4	1.0000	1.0000
	TV1C8.2	Group 1 vs Group 2	0.2359	0.6563
		Group 1 vs Group 3	0.6730	0.9229
		Group 1 vs Group 4	0.2766	0.6988
		Group 2 vs Group 3	0.4894	0.8805
		Group 2 vs Group 4	0.8633	0.9808
		Group 3 vs Group 4	0.3401	0.7682
Blocking	CD4 to A244D11gp120	Group 1 vs Group 2	**0.0002**	**0.0105**
		Group 1 vs Group 3	0.6244	0.9081
		Group 1 vs Group 4	0.9416	0.9936
		Group 2 vs Group 3	**0.0056**	0.0832
		Group 2 vs Group 4	**0.0077**	0.1049
		Group 3 vs Group 4	0.7115	0.9263
	A32 to A244D11gp120	Group 1 vs Group 2	0.1086	0.4842
		Group 1 vs Group 3	0.4947	0.8805
		Group 1 vs Group 4	0.4088	0.8351
		Group 2 vs Group 3	0.6507	0.9187
		Group 2 vs Group 4	0.0895	0.4239
		Group 3 vs Group 4	0.1821	0.6359
	2G12 to A244D11gp120	Group 1 vs Group 2	**0.0037**	0.0790
		Group 1 vs Group 3	0.9104	0.9936
		Group 1 vs Group 4	0.7430	0.9263
		Group 2 vs Group 3	0.2224	0.6563
		Group 2 vs Group 4	0.2973	0.7048
		Group 3 vs Group 4	1.0000	1.0000
	CH58 to A244D11gp120	Group 1 vs Group 2	0.9446	0.9936
		Group 1 vs Group 3	0.2872	0.7048
		Group 1 vs Group 4	**0.0001**	**0.0079**
		Group 2 vs Group 3	0.5052	0.8805
		Group 2 vs Group 4	**0.0005**	**0.0190**
		Group 3 vs Group 4	**0.0000**	**0.0079**
	PG9 to A244D11gp120	Group 1 vs Group 2	0.9626	0.9936

		Group 1 vs Group 3	0.9626	0.9936
		Group 1 vs Group 4	0.7430	0.9263
		Group 2 vs Group 3	0.4363	0.8726
		Group 2 vs Group 4	0.8821	0.9859
		Group 3 vs Group 4	0.5457	0.8805
	CH01 to A244D11gp120	Group 1 vs Group 2	0.2359	0.6563
		Group 1 vs Group 3	0.7972	0.9507
		Group 1 vs Group 4	0.7626	0.9386
		Group 2 vs Group 3	0.2973	0.7048
		Group 2 vs Group 4	0.7962	0.9507
		Group 3 vs Group 4	0.5613	0.8980
	CD4 to B6240D11gp120	Group 1 vs Group 2	0.1388	0.5330
		Group 1 vs Group 3	0.8341	0.9649
		Group 1 vs Group 4	0.6058	0.9017
		Group 2 vs Group 3	0.2679	0.6859
		Group 2 vs Group 4	0.3401	0.7682
		Group 3 vs Group 4	0.6665	0.9206
	CH01 to 902114B2gp140	Group 1 vs Group 2	0.6458	0.9185
		Group 1 vs Group 3	0.5767	0.9017
		Group 1 vs Group 4	0.5042	0.8805
		Group 2 vs Group 3	0.8485	0.9756
		Group 2 vs Group 4	0.5704	0.9017
		Group 3 vs Group 4	0.6343	0.9157
	PG9 to B6240D11gp120	Group 1 vs Group 2	0.2652	0.6859
		Group 1 vs Group 3	0.2359	0.6563
		Group 1 vs Group 4	0.4807	0.8805
		Group 2 vs Group 3	0.7299	0.9263
		Group 2 vs Group 4	0.1543	0.5697
		Group 3 vs Group 4	0.0892	0.4239
ADCC	1086Cgp140	Group 1 vs Group 2	0.2574	0.6859
		Group 1 vs Group 3	**0.0123**	0.1384
		Group 1 vs Group 4	**0.0137**	0.1434
		Group 2 vs Group 3	0.2615	0.6859
		Group 2 vs Group 4	0.1964	0.6387
		Group 3 vs Group 4	1.0000	1.0000
	A244D11gp120	Group 1 vs Group 2	0.1521	0.5697
		Group 1 vs Group 3	0.1238	0.5166
		Group 1 vs Group 4	**0.0097**	0.1237
		Group 2 vs Group 3	0.7823	0.9507
		Group 2 vs Group 4	0.1312	0.5330
		Group 3 vs Group 4	0.2941	0.7048
	CAP174D11gp120	Group 1 vs Group 2	0.3575	0.7980
		Group 1 vs Group 3	**0.0027**	0.0652
		Group 1 vs Group 4	0.2660	0.6859
		Group 2 vs Group 3	**0.0196**	0.1712
		Group 2 vs Group 4	0.5887	0.9017
		Group 3 vs Group 4	0.2252	0.6563

	CAP260D11gp120	Group 1 vs Group 2	0.5370	0.8805
		Group 1 vs Group 3	0.3849	0.7980
		Group 1 vs Group 4	0.6458	0.9185
		Group 2 vs Group 3	0.7149	0.9263
		Group 2 vs Group 4	1.0000	1.0000
		Group 3 vs Group 4	0.7719	0.9440
	CH505TFD7gp120	Group 1 vs Group 2	0.2210	0.6563
		Group 1 vs Group 3	0.9431	0.9936
		Group 1 vs Group 4	0.4946	0.8805
		Group 2 vs Group 3	0.2120	0.6563
		Group 2 vs Group 4	0.5033	0.8805
		Group 3 vs Group 4	0.6186	0.9081
	Ko244D11gp120	Group 1 vs Group 2	0.0771	0.3897
		Group 1 vs Group 3	**0.0283**	0.2324
		Group 1 vs Group 4	0.1602	0.5803
		Group 2 vs Group 3	0.8834	0.9859
		Group 2 vs Group 4	0.7895	0.9507
		Group 3 vs Group 4	0.5923	0.9017

Table S2. Sera neutralization (ID_{50}) of tier 1 and 2 Envelopes two weeks pre-challenge.

	SVA-MLV	TH023.6	96ZM651.2	TV1C8.2	CM244.c01	Ce1086_B2	CAP174.1.06_F3_1B	CAP260.2.00_TA1_1B	Ko224_T87_2_4	SHIV CH505.375H.dCT/RhPBMC
	Neg Ctrl	CRF01_AE	Clade c	Clade C	CRF01_AE	Clade C	Clade C	Clade C	Clade C	Clade C
Group	Tier 2	Tier 1	Tier 1	Tier 1	Tier 2	Tier 2	Tier 2	Tier 2	Tier 2	Chellenge Srain
	<20	**1,281**	<20	**904**	<20	<20	<20	<20	<20	<20
	<20	**2,581**	<20	**652**	<20	<20	<20	<20	<20	<20
	<20	**4,419**	**23**	**1,297**	<20	<20	<20	<20	<20	<20
Group 1:	<20	**4,240**	<20	**1,450**	<20	<20	<20	<20	<20	**20**
Pantavalent_ECCCC	<20	**5,758**	**25**	**2,216**	<20	<20	<20	**29**	<20	<20
	<20	**2,590**	<20	**1,451**	<20	<20	<20	<20	<20	<20
	<20	**10,115**	<20	**2,623**	<20	<20	<20	<20	<20	**35**
	<20	**4,256**	<20	**3,637**	<20	<20	<20	**21**	<20	<20
	<20	**576**	<20	**647**	<20	<20	<20	<20	<20	<20
	<20	**103**	<20	**1,589**	<20	<20	<20	<20	<20	<20
	<20	**2,576**	<20	**6,349**	<20	<20	<20	<20	<20	**27**
Group 2:	<20	**433**	<20	**14,508**	<20	<20	<20	<20	<20	**35**
Bivalent_CC	<20	**2,709**	<20	**1,457**	<20	<20	<20	<20	<20	<20
	<20	**700**	<20	**1,500**	<20	<20	<20	<20	<20	<20
	<20	**527**	<20	**1,101**	<20	<20	<20	<20	<20	<20
	<20	**517**	<20	**4,539**	<20	<20	<20	<20	<20	<20
	<20	**432**	<20	**3,736**	<20	<20	<20	<20	<20	<20
	<20	**10,801**	<20	**908**	<20	<20	<20	<20	<20	<20
	<20	**754**	<20	**1,473**	<20	<20	<20	<20	<20	<20
	<20	**2,237**	<20	**895**	<20	<20	<20	<20	<20	**20**
Group 3:	<20	**4,127**	<20	**1,720**	<20	<20	<20	<20	<20	<20
Bivalent_EC	<20	**952**	<20	**439**	<20	<20	<20	<20	<20	<20
	<20	**13,285**	<20	**5,977**	<20	<20	<20	<20	<20	**30**
	<20	**2,708**	**24**	**2,869**	<20	<20	<20	<20	<20	**21**
	<20	**5,070**	<20	**2,307**	<20	<20	<20	<20	<20	**21**
	<20	**3,581**	<20	**3,029**	<20	<20	<20	<20	<20	**21**
	<20	**18,237**	<20	**4,696**	<20	<20	<20	<20	<20	<20
	<20	**6,268**	**22**	**3,331**	<20	<20	<20	**59**	**20**	**30**
	<20	**4,462**	<20	**2,383**	<20	<20	<20	<20	<20	**33**
Group 4:	<20	**2,972**	<20	**2,308**	<20	<20	<20	<20	<20	<20
Trivalent_CCC	<20	**441**	<20	**588**	<20	<20	<20	<20	<20	<20
	<20	**162**	<20	**255**	<20	<20	<20	<20	<20	<20
	<20	**4,687**	<20	**6,756**	<20	<20	<20	**35**	<20	**26**
	<20	**1,754**	**25**	**1,907**	<20	<20	<20	<20	<20	<20
	<20	**5,472**	<20	**7,469**	<20	<20	<20	<20	<20	<20

Table S3. Hazard ratios for primary outcomes at time of challenge (week 143) using a pairwise cox proportional hazard model.

Immune marker	Antigen	Groups included	Hazard Ratio (95% CI)	P-value
**IgG binding antibodies**	CH505TFD7gp120	1 and control	**0.615 (0.399-0.949)**	**0.0281**
		4 and control	**0.546 (0.341-0.872)**	**0.0114**
**V1V2 AUC-MB**	ALL	1 and control	**0.561 (0.324-0.971)**	**0.0389**
		4 and control	**0.529 (0.296-0.944)**	**0.0312**
**ADCC**	CH505TFD7gp120	1 and control	**0.610 (0.431-0.862)**	**0.0050**
		4 and control	**0.693 (0.518-0.926)**	**0.0133**
**ADCP**	CH505TFD7gp120	1 and control	**0.927 (0.861-0.998)**	**0.0434**
		4 and control	**0.906 (0.838-0.980)**	**0.0135**
**CD4 Blocking**	A244D11gp120	1 and control	0.0001 (0-1.76×10^{23})	0.7641
		4 and control	0.0002 (0-1.42×10^{9})	0.5788
